# Clinical, Genomic, and Pharmacological Study of *MYCN*-Amplified *RB1* Wild-Type Metastatic Retinoblastoma

**DOI:** 10.3390/cancers12092714

**Published:** 2020-09-22

**Authors:** Santiago Zugbi, Daiana Ganiewich, Arpita Bhattacharyya, Rosario Aschero, Daniela Ottaviani, Claudia Sampor, Eduardo G. Cafferata, Marcela Mena, Mariana Sgroi, Ursula Winter, Gabriela Lamas, Mariona Suñol, Manuel Daroqui, Edgardo Baialardo, Beatriz Salas, Anirban Das, Adriana Fandiño, Jasmine H. Francis, Fabiana Lubieniecki, Cinzia Lavarino, Ralph Garippa, Osvaldo L. Podhjacer, David H. Abramson, François Radvanyi, Guillermo Chantada, Andrea S. Llera, Paula Schaiquevich

**Affiliations:** 1 Precision Medicine, Hospital de Pediatría JP Garrahan, Buenos Aires 1245, Argentina; santiagozugbi@gmail.com (S.Z.); dganiewich@leloir.org.ar (D.G.); rosarioaschero@gmail.com (R.A.); eemena@garrahan.gov.ar (M.M.); gchantada@garrahan.gov.ar (G.C.); 2 National Scientific and Technical Research Council, CONICET, Buenos Aires 1425, Argentina; ecafferata@leloir.org.ar (E.G.C.); opodhajcer@leloir.org.ar (O.L.P.); allera@leloir.org.ar (A.S.L.); 3 Laboratory of Molecular and Cellular Therapy, Instituto Leloir-Instituto de Investigaciones Bioquímicas de Buenos Aires (IIBBA), Buenos Aires 1405, Argentina; 4 Department of Paediatric Haematology and Oncology, Tata Memorial Hospital, Kolkata 700160, India; arpita.bhattacharyya@tmckolkata.com (A.B.); anirban.das@tmckolkata.com (A.D.); 5 Institut Curie; PSL Research University, Centre National de la Recherche Scientifique (CNRS), UMR144, Equipe Ligue Contre le Cancer, 75005 Paris, France; daniela.ottaviani@curie.fr (D.O.); francois.radvanyi@curie.fr (F.R.); 6 Hematology-Oncology Service, Hospital de Pediatría JP Garrahan, Buenos Aires 1245, Argentina; claudiasampor@hotmail.com; 7 Ophthalmology Service, Hospital de Pediatría JP Garrahan, Buenos Aires 1245, Argentina; msgroi@garrahan.gov.ar (M.S.); afandino@garrahan.gov.ar (A.F.); 8 Pathology Service, Hospital de Pediatría JP Garrahan, Buenos Aires 1245, Argentina; winter.u.a@gmail.com (U.W.); gslamas1@gmail.com (G.L.); flubieniecki@garrahan.gov.ar (F.L.); 9 Pathology Service, Hospital Sant Joan de Deu, 08950 Barcelona, Spain; MSunol@hsjdbcn.org; 10 Cytogenetics Service, Hospital de Pediatría JP Garrahan, Buenos Aires 1245, Argentina; jdaroqui@garrahan.gov.ar (M.D.); ebaialardo@garrahan.gov.ar (E.B.); 11 Paediatric Oncology Service, Hospital Asencio Villaroel, Cochabamba 2500, Bolivia; bett700713@yahoo.es; 12 Ophthalmic Oncology Service, Memorial Sloan-Kettering Cancer Center, New York, NY 10065, USA; francij1@mskcc.org (J.H.F.); Abramsod@mskcc.org (D.H.A.); 13 Pediatric Hematology and Oncology Service, Hospital Sant Joan de Deu, 08950 Barcelona, Spain; clavarino@sjdhospitalbarcelona.org; 14 Laboratory of Molecular Oncology, Hospital Sant Joan de Déu, Fundación Sant Joan de Déu, 08950 Barcelona, Spain; 15 Gene editing and screening core facility, Department of Cancer Biology and Genetics, Memorial Sloan-Kettering Cancer Center, New York, NY 10065, USA; garippar@mskcc.org

**Keywords:** metastatic retinoblastoma, *MYCN* amplification, wild-type *RB1*, orbital dissemination

## Abstract

**Simple Summary:**

We present two cases of a subtype of retinoblastoma, a rare ocular tumor in children, presenting without the typical mutation in the RB1 gene but showing amplification of the transcription factor MYCN frequently reported in pediatric malignancies. Even though previous reports suggested that this tumor sybtype could present metastases, patients with metastatic disease were not reported. Our cases had metastasis to the orbit and lymph nodes and poor sensitivity to standard chemotherapy but no dissemination to the central nervous system, as described for patients with mutations in the RB1. We were able to grow the cells of one of our patients in vitro, perform comprehensive genomic analysis that showed previously not reported mutations and other chromosomal alterations. In an animal model, we could reproduce the clinical dissemination and we identified an innovative active drug combination that could help for the treatment of these children with poor prognosis.

**Abstract:**

An uncommon subgroup of unilateral retinoblastomas with highly aggressive histological features, lacking aberrations in *RB1* gene with high-level amplification of *MYCN* (*MCYN*${}_{ampl}$*RB1*${}^{+/+}$) has only been described as intra-ocular cases treated with initial enucleation. Here, we present a comprehensive clinical, genomic, and pharmacological analysis of two cases of *MCYN*${}_{ampl}$*RB1*${}^{+/+}$ with orbital and cervical lymph node involvement, but no central nervous system spread, rapidly progressing to fatal disease due to chemoresistance. Both patients showed in common *MYCN* high amplification and chromosome 16q and 17p loss. A somatic mutation in *TP53*, in homozygosis by LOH, and high chromosomal instability leading to aneuploidy was identified in the primary ocular tumor and sites of dissemination of one patient. High-throughput pharmacological screening was performed in a primary cell line derived from the lymph node dissemination of one case. This cell line showed resistance to broad spectrum chemotherapy consistent with the patient’s poor response but sensitivity to the synergistic effects of panobinostat–bortezomib and carboplatin–panobinostat associations. From these cells we established a cell line derived xenograft model that closely recapitulated the tumor dissemination pattern of the patient and served to evaluate whether triple chemotherapy significantly prolonged survival of the animals. We report novel genomic alterations in two cases of metastatic *MCYN*${}_{ampl}$*RB1*${}^{+/+}$ that may be associated with chemotherapy resistance and in vitro/in vivo models that serve as basis for tailoring therapy in these cases.

## 1. Introduction

The majority of retinoblastoma cases are initiated by the biallelic inactivation of the RB1 gene (*RB1*${}^{-/-}$) [1,2,3,4,5]. Fewer than 2% of retinoblastomas lack aberrations in RB1 but display high-level amplification of *MYCN*. To date, only patients with intraocular disease *MYCN* amplified and *RB1* wild-type patients (*MYCN*${}_{ampl}$*RB1*${}^{+/+}$) have been reported [6]. The reported cases include young patients (median age at diagnosis of 4.5 months) with unilateral disease and characteristic histopathological features (undifferentiated cells with prominent nucleoli, signs of necrosis, and little calcification). Even though tumor aggressiveness has been suspected based on its clinical and histopathological features, no metastatic case has yet been reported. Thus, it remains unknown if *MYCN*${}_{ampl}$*RB1*${}^{+/+}$ metastatic disease shares biological features similar to intraocular cases and if treatment strategies useful for *RB1*${}^{-/-}$ disease are effective.

To the best of our knowledge, this is the first report of two cases of metastatic *MYCN*${}_{ampl}$*RB1*${}^{+/+}$ retinoblastomas. We report the clinical, histopathological, genomic, and pharmacological features of these patients, and provide information about the characteristics that are related to this highly aggressive tumor and clinical management.

## 2. Results

### 2.1. Report of Cases

#### 2.1.1. Case 1

**Figure 1:**
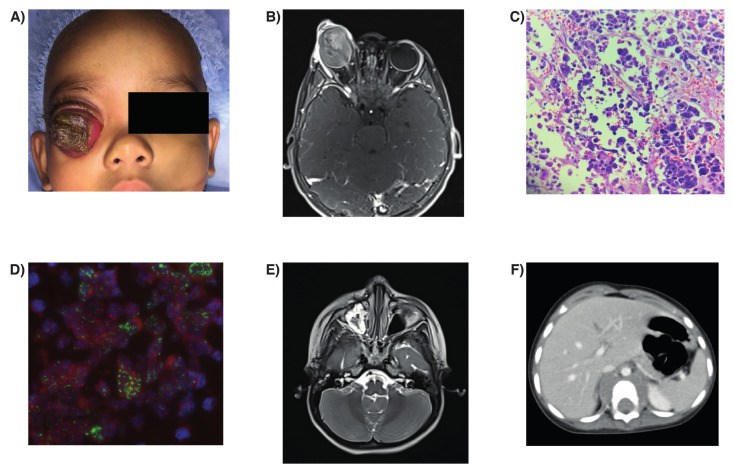
(**A**) Clinical presentation of Patient 1 denoting a fungating mass in the right eye; (**B**) T2-weighted axial MRI at hospitalization showing a gross orbital mass without radiological involvement of the optic nerve and no intracranial extension or metastasis; (**C**) Histopathological findings of the orbital tissue after exenteration. Hematoxylin & eosin stain showing pleomorphic cells, anaplasia, and areas of necrosis; (**D**) Fluorescence in situ hybridization (FISH) of *MYCN* showing amplification of this gene (red signals) with respect to AFF3 (green signals) as a reference. Original magnification 20x; (**E**) T2-weighted axial MRI after multimodal treatment, including exenteration, adjuvant chemotherapy, and radiotherapy showing a relapsing mass in the maxillary sinus and (**F**) a paravertebral mass with intraspinal extension.

A 30-month-old child without family history of retinoblastoma presenting with a fungating orbital mass emanating from the right eye (Figure 1A) was admitted to the Tata Medical Center, Kolkata, India. The child presented a one-year history of leukocoria and a progressive proptosis evolving to an orbital mass over the last eight months. The fellow eye was normal. Magnetic resonance imaging (MRI) of the orbit and brain at diagnosis is shown in Figure 1B.

Bone marrow aspirates and biopsies and cerebrospinal fluid (CSF) examination were negative for tumor cells. The patient was diagnosed as Stage III retinoblastoma according to the International Retinoblastoma Staging System and started treatment with vincristine (0.05 mg/kg/day), carboplatin (18.7 mg/kg/day), and etoposide (5 mg/kg/day). However, the patient demonstrated progression on that chemotherapy combination, so orbital exenteration was performed, followed by orbital radiotherapy at a dose of 45 Gy over 25-fractions. Initial biopsy of the exenterated orbital mass revealed a poorly differentiated tumor, markedly pleomorphic cells, lack of rosettes, severe anaplasia, calcification, and large areas of necrosis (Figure 1C). The optic nerve was free of tumor along its extension. *MYCN* amplification was detected by fluorescence in vitro hybridization (FISH) (Figure 1D). Positive staining of p53 was observed in 70% of nuclei of the tumor cells (Figure S1A).

Following completion of radiation, the patient presented with a mass in the maxillary sinus (Figure 1E), paraparesis, and diffuse swelling over the right cheek and the computed tomography (CT) showed a paravertebral soft tissue mass at D11 with anterior epidural soft tissue compressing the anterior thecal sac and bilateral foramina (Figure 1F). MRI of the brain revealed orbital disease without evidence of intracranial involvement (Figure 1E). The patient received palliative care and died within a few weeks.

#### 2.1.2. Case 2

The child was diagnosed with unilateral retinoblastoma (Group D) at the age of 17 months with no family history, and treated in Bolivia with seven cycles of standard carboplatin (18 mg/kg/day), etoposide (5 mg/kg/day) and vincristine (0.05 mg/kg/day) chemoreduction. Intraocular tumor progression was noted and the eye was enucleated. However, three months after surgery, an orbital relapse and lymph node suspicious for metastasis was found, and the family sought care at our institution.

**Figure 2:**
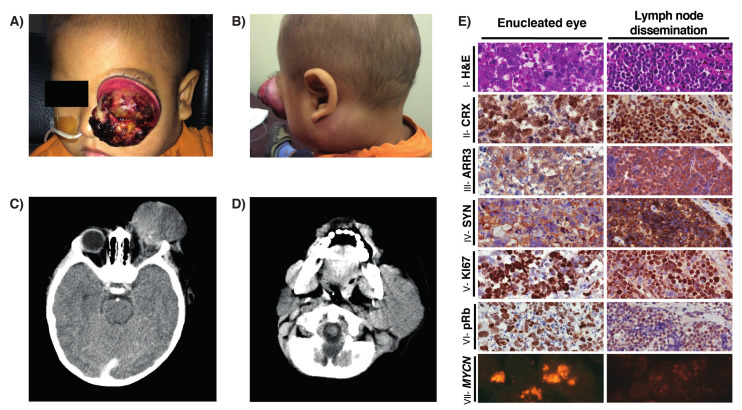
(**A**,**B**) Photograph of Patient 2 showing an exuberant left orbital mass correspondent with unilateral retinoblastoma and homolateral cervical lymph node extension; (**C**,**D**) Axial CT scan showing a large intra and extra-orbital mass with homolateral (left side) cervical lymphadenopathy; (**E**) Histology of the orbital mass and lymph node biopsy after neoadjuvant chemotherapy. Left column, primary tumor; right column lymph node dissemination immunostainings; (I) Hematoxylin & eosin stain showing pleomorphic and large undifferentiated cells with prominent nucleoli, and hyperchromatic nuclei. Positive stain for (II) cone-rod homebox transcription factor (CRX), (III) late cone photoreceptor marker arrestin3 (ARR3), (IV) neuronal marker synaptophysin (SYN), (V) proliferation marker Ki67, and (VI) retinoblastoma protein. (VII) FISH analysis showing amplification of *MYCN* (2p24.3; spectrum red) Original magnification 20x.

Clinical examination showed a left orbital mass and gross multiple cervical and submaxillary lymph nodes (Figure 2A,B). CT scan of the brain and neck showed a large intra and extra-orbital mass with left cervical lymphadenopathy, but no intracranial dissemination (Figure 2C,D). Bilateral bone marrow aspirates and biopsies and CSF specimen were negative for tumor cells and minimally disseminated disease while using real time q-PCR for cone-rod homebox (CRX) mRNA [7,8]. Pathological review of the enucleated eye at our institution, the orbital, and the lymph node biopsies showed large and pleomorphic cells with hyperchromatic nuclei, prominent nucleoli, and lack of rosettes (Figure 2E). Besides, an extended immunohistochemical panel showed that both samples were positive for CRX and arrestin3 photoreceptor markers, retinoblastoma protein, synaptophysin and >75% of positive cells for Ki67. *MYCN* amplification was evidenced by FISH (Figure 2E). Additionally, both the primary ocular tumor and lymph node were positive for p53 in almost 100% of nuclei of the tumor cells (Figure S1B,C).

This patient was included in a prospective protocol for procurement of biological material for genomic studies and cell cultures for metastatic disease which was approved by the Institutional Review Board at Hospital de Pediatria JP Garrahan (protocol #838). Written informed consent was obtained from parents. An orbital and lymph node tumor biopsy was performed in order to confirm the diagnosis. Specimens from the enucleated eye became available at a later time.

Intensive multi-agent chemotherapy was given according to the COG-ARET 0321 protocol consisting of vincristine (0.05 mg/kg/day), cisplatin (3.5 mg/kg/day), cyclophosphamide (65 mg/kg/day), and etoposide (4 mg/kg/day). Because of disease progression after the first two cycles, palliative orbital and cervical radiotherapy (21-Gy) along with oral temozolomide 80 mg/kg/day, and etoposide 20mg/kg/day were given. Nonetheless, after 10 weeks the patient died with progressive disease. A limited autopsy showed tumoral infiltration of multiple supra- and infra-diaphragmatic lymph nodes with tumoral implants in the diaphragm and peri-renal fat as well as massive liver metastasis involving more than 95% of the liver parenchyma. Material from the central nervous system (CNS) could not be obtained due to lack of consent.

### 2.2. Genomic Analysis

In both patients Sanger sequencing and copy number analysis by Multiplex Ligation-dependent Probe Amplification (MLPA) of the normal tissue and the enucleated eye showed no alterations in *RB1*. In patient 2, the availability of samples from the lymph node dissemination allowed for us to confirm the lack of RB1 promoter hypermethylation in this specimen.

**Figure 3:**
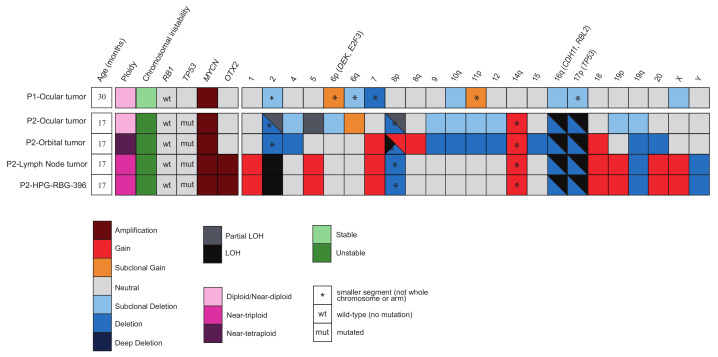
Each row represents individual samples and the rectangular boxes correspond to the status of each of the main characteristics depicted. Boxes are partitioned if more than one relevant feature coexists. Patient 1 (P1) and Patient 2 (P2) ocular tumors were analyzed with Oncoscan array and Sanger sequencing, while P2 metastases were studied by WES. Ploidy is normal for P1 ocular tumor, whereas it is abnormal in P2, even in the ocular tumor in which a distinction between tetraploid or diploid was not possible. *TP53* and *RB1* somatic mutations are depicted along with focal amplifications on *MYCN* and *OTX2*. Each number on top of the boxes represents a chromosome or chromosome arm where at least a single CNA has been found. Relevant retinoblastoma driver genes are between brackets. Copy number gains are shown in red, and blue represents losses, while the intensity of the color shade is proportional to the value of the log-ratio (LRR).

Copy number alteration (CNA) analysis from the tumor of Patient 1 using Oncoscan showed a diploid genome with a high-level focal amplification (>20 copies) of the 2p24.2 region harboring the *MYCN* gene (Figure 3 and Figure S2). Gains in chromosome 6p and 11q, and losses in 6q, 7q (LOH), and the X chromosome were also evident. In a low (20%) subclonal level, losses of chromosome 10, 16q, and 17p arms were detected. No alterations were found in chromosome 13 that harbors *RB1*. No mutation was observed in *TP53* gene by Sanger sequencing.

CNA analysis of the primary ocular tumor sample from Patient 2 using Oncoscan showed a high-level (>60 copies) 2p focal amplification harboring *MYCN* and no apparent copy number alterations in chromosome 13. In addition, we observed clonal loss in 16q (spanning both *CDH11* and *RBL2*) and in 17p (including the *TP53* gene) (Figure 3). Chromosome Analysis Software estimated a ploidy of 2. However, the overall log-ratio (LRR) and B-allele frequency (BAF) profiles of the primary ocular tumor were highly heterogeneous with no single dominant copy number profile, evidence that suggested aneuploidy. For this reason, a near-tetraploid state (that would be indistinguishable from diploid in this analysis) could not be ruled out (Figure S3).

For this patient, a specimen from the lymph node metastasis was used to establish a primary cell culture designated as HPG-RBG1. The genetic background of these cells was determined by the identical STR profile to that found in the paired tumor sample (Table S1). Sanger and MLPA analysis of *RB1* showed no deletions, gains, losses, or LOH in this gene. In vitro, these cells showed a growth pattern as tumorspheres and morphological and immunohistochemical features that were comparable to the matched tumor (Figure S4A,B) [9,10]. The expression and functional status of pRb in HPG-RBG1 cells was validated by western blot and immunoprecipitation (Figure S4C). GD2 ganglioside staining by immunofluorescence was evident in these cells (Figure S4D).

Consistent with the analysis of the primary tumor, CNAs detected by whole exome sequencing (WES) of the orbital and lymph node metastasis and HPG-RBG1 cells using Control-FREEC revealed highly heterogeneous LRR and BAF profiles, suggesting aneuploidy (Figure S5). Karyotype analysis of HPG-RBG1 showed several numerical and structural chromosomal abnormalities, though all of the metaphases analyzed present a modal number close to triploidy. We also detected chromosome 1 and chromosome 7 in tetra or even pentasomy as well as homogeneous staining regions (HRS) and double-minutes (dmin), typically seen in *MYCN* amplified cells. These findings and other features are depicted in (Figure S6). Based on these observations and manual review of LRR and BAF profiles, we decided to manually classify lymph node metastasis and HPG-RBG1 samples as near-triploid and the orbital metastasis sample as near-tetraploid.

The interpretation of data when considering ploidy status showed again the focal high-level (>60x) amplification of *MYCN* in 2p and the lack of an *RB1* deletion or LOH in both metastatic sites (Figure 3 and Figure S5). They also shared gains of 7 and 18 whole chromosomes and a segment of 14q, along with a loss of 8p, 19q and the deletion in LOH of 16q and 17p arms, including *CDH11*, *RBL2*, and *TP53* genes.

Only the lymph node metastasis and HPG-RBG1 cells showed a high-level amplification (>20×) in 14q harboring *OTX2* (a gene that has been described as amplified in retinoblastoma [11]), as well as other focal gains or amplifications in 2q, 11q and 14q that did not include cancer-related genes. Gains in chromosomes 1, 5, 18, 20 and X and 19p arm together with the loss of chromosome Y were also evident only in lymph node samples. Chromosome 2 was found in copy-neutral LOH, a feature that is also present in a subclonal fashion in the primary ocular tumor (Figure S5).

On the other hand, only orbital metastasis had a gain in chromosome 8, except for the deleted segment at the beginning of 8p, which was found in LOH in this specimen and in a subclonal LOH in the primary tumor. Losses of chromosomes 2, 4, 9, 12, 15, 20 and 10q, 11p arms were also evident, most of which were seen as subclonal in the primary tumor.

Of note, there was no evidence of extra gains in chromosome 6 in any of the samples and therefore in the harbored driver genes *DEK* and *E2F3*.

WES analysis revealed a *TP53* mutation ( nM_000546.4:c.713G>A, p.(Cys238Tyr)) with an allele frequency of 69% and 98% in metastatic orbital metastasis and lymph node metastasis, respectively. In both cases, the allele frequency resulted from the aforementioned LOH in chromosome 17; a lower allele frequency in orbital metastasis was explained by normal cell contamination, according to the tumor purity estimation. This *TP53* mutation was then identified by Sanger sequencing in the primary ocular tumor, but, due to lack of availability of this sample for WES analysis, we could not estimate the allele frequency. This mutation is labeled as likely pathogenic in ClinVar [12] and as pathogenic in Varsome [13]. Besides, an intense immunohistochemical p53 staining of all tumor cells was detected in these tumor samples (Figure S1B,C).

Several other potentially deleterious somatic mutations in the specimens from the dissemination sites of Patient 2 were identified by WES. A missense variant in *TSC1* tumor suppressor gene and a truncating variant in the *BFSP2* gene were detected only in the orbital metastasis. In the lymph node metastasis and HPG-RBG1 samples, three truncating variants in *LRFN1*, *MTF1*, and *TAF7* were identified all being novel genetic alterations in retinoblastoma (see Figure 3 and Table S2 for the complete list of potentially damaging variants). However, no alterations were encountered in recurrently mutated genes in *RB1*${}^{-/-}$ retinoblastoma, such as *BCOR* or *CREBBP* [2,5,14,15].

### 2.3. In Vitro and In Vivo Pharmacological Sensitivity

In order to identify compounds active for this tumor subtype, we performed a large-scale high-throughput screening (HTS) and compared to the sensitivity of patient two-derived cell line HPG-RBG1 with Y79 (commercial cell line with *RB1*${}^{-/-}$ and *MYCN* amplification) and HPG-RBT-12L (derived from an untreated human intraocular tumor with no *MYCN* amplification [16]). From the primary screen, HTS assay diagnostics showed to be robust as the mean (SD) Z’ factor of the screened plates was 0.62 (0.02), 0.49 (0.07), and 0.56 (0.05) for Y79, HPG-RBG1, and HPG-RBT-12L cells, respectively. Thus, all of the screened plates were included in the analysis.

**Figure 4:**
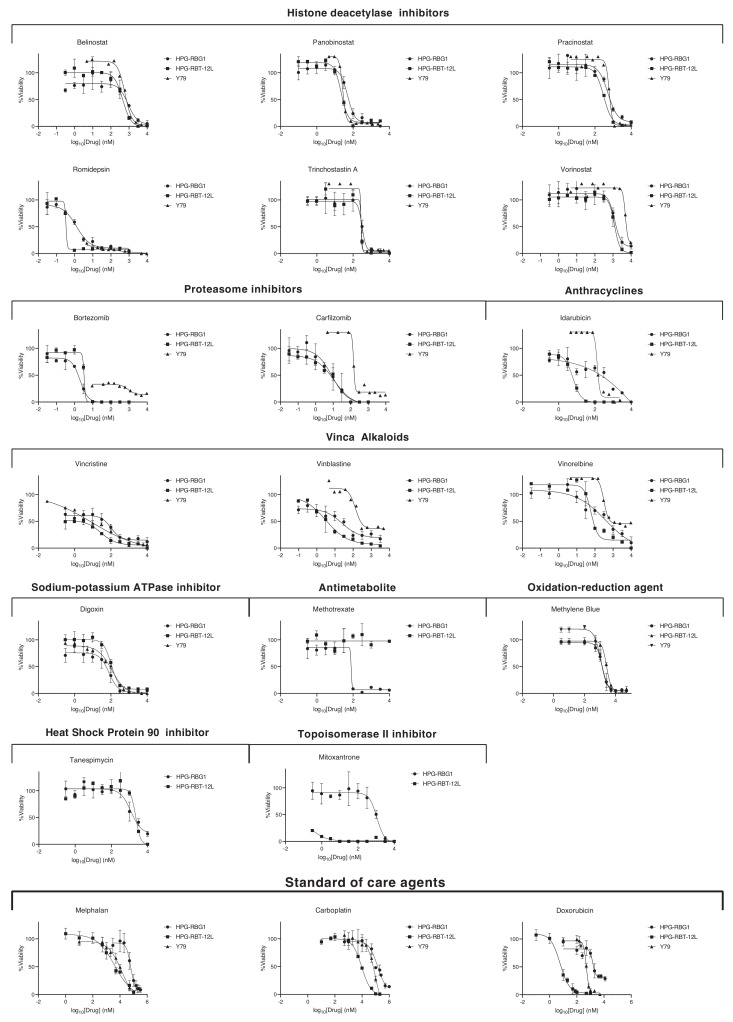
Tumor cell growth inhibition of 17 compounds in HPG-RBG1, HPG-RBT-12L, and Y79 cells. The assay was performed in 384-well plates and cells were exposed to 10 increasing concentrations (0.03 nM-9.6 μM) and cell viability was determined at 72 h using CellTiter-Glo. Symbols represent percentage of cell proliferation as compared to untreated control cells, expressed as means (SEM) of three independent experiments. The data were fitted using a four-parameter non-linear regression by Graphpad Prism and the derived metrics are described in detailed in Table S4. Dose-response curves for standard-of-care agents were manually performed and are depicted at the bottom row.

A total of 128 (5%), 109 (4%), and 77 (3%) hits were active against HPG-RBT-12L, Y79, and HPG-RBG1 cells, respectively, as these agents showed > 80% cell growth inhibition at the maximum screened concentration of 4.8 μM (Table S3). From the set of hits active in HPG-RBG1 cells, we filtered for those compounds that are currently used in the clinics of pediatric tumors or show evidence of Phase I/II clinical trials in children and are commercially available for clinically feasible translation (full description of selection criteria in Figure S7). In total, 17 drugs were further screened, and full dose-response curves were performed in order to calculate the concentration that results in a 50% decrease in tumor cell growth or EC50 for the three cell lines (Figure 4, Table S4). Identified active drug classes (number of drugs) included histone deacetylase inhibitors (6 drugs, 35% of the total selection), proteasome inhibitors (2), heat shock protein inhibitors (1), cardiac glycosides (1), topoisomerase II inhibitors (1), oxidation-reduction agent/dye (1), antimetabolite (1), and only three drugs that belong to the vinca alkaloids (3) and anthracyclines (1) groups that are currently used for the treatment of retinoblastoma.

**Figure 5:**
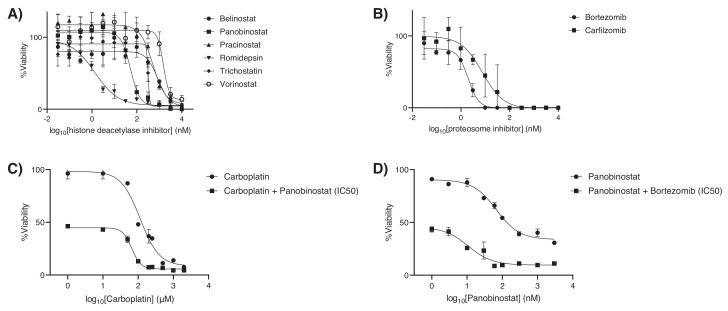
(**A**) Dose-response curves for histone deacetylase and (**B**) proteasome inhibitors. (**C**) Dose-dependent cytotoxicity of carboplatin as a single-agent and in combination with panobinostat at the EC50 (67.4 nM), and (**D**) panobinostat in combination with bortezomib at the IC50 (5.2 nM). Addition of panobinostat at 67.4 nM potentiated the cytotoxic effect of carboplatin by shifting the EC50 of carboplatin from 115 μM to 65 μM, whereas the addition of bortezomib 5.2 nM shifted the EC50 of panobinostat from 67.4 nM to 10.8 nM. Symbols represent % of cell proliferation as compared to untreated control cells, expressed as means (SEM) of three independent experiments.

Subsequently, combinatorial screening was performed on panobinostat and bortezomib with carboplatin to potentiate its cytotoxic effects. Other standard of care drugs were not eligible (e.g., topotecan and doxorubicin) as the EC50s were too high to be achieved in vivo. Six drugs of the HDAC inhibitor group were active against HPG-RBG1 (Figure 5), but only panobinostat was selected for further synergistic evaluations as it has already been tested as part of combination treatments in preclinical models of pediatric tumors and published pharmacokinetic data supports that the EC50 (67.2 nM) would be clinically attainable [17,18,19,20].

The exposure of HPG-RBG1 to increasing concentrations of panobinostat in combination with bortezomib at the EC50 (5.2 nM, 95%CI: 4.2–6.4) potentiated the cytotoxic effect of panobinostat by shifting eight-fold the EC50 from 67 nM to 8 nM with a combination index <1. Moreover, the combination of carboplatin and panobinostat at the EC50 also resulted in synergistic activity (combination index <1) and a reduction of carboplatin EC50 from 115 μM to 65 μM (Figure 5). The fraction of cells affected by each drug combination evaluated in the study is depicted in Table S5.

**Figure 6:**
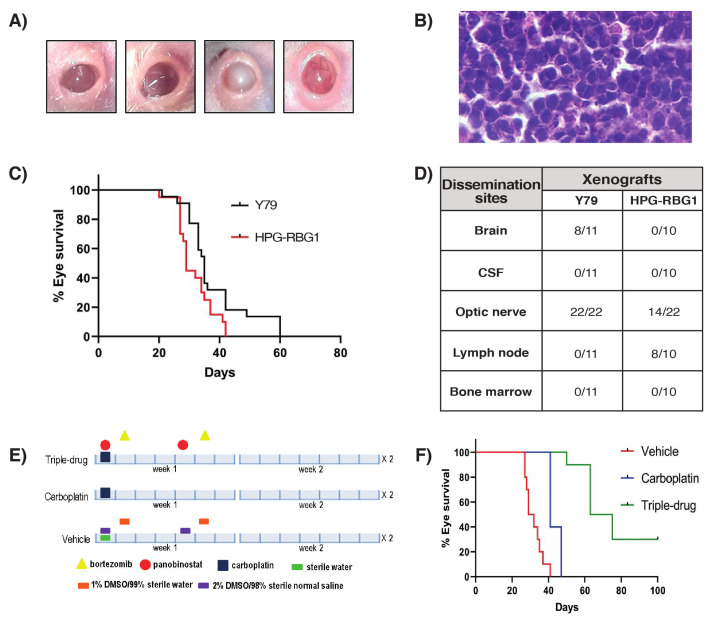
(**A**) Macroscopic assessment of tumor growth after intravitreal injection of HPG-RBG1 cells to immunodeficient mice. Progressive stages of tumor growth from stage 0 corresponding to the normal mouse eye to stage 1 showing small tumors in the posterior segment, stage 2 with the presence of tumor filling the full posterior segment and increase in the eye size, and stage 3 with eyes three-fold the normal size and proptosis; (**B**) Hematoxylin & eosin of the CDXs eye showing the same characteristics (large, anaplastic cells) as those observed for the orbital tumor of Patient 2 (40x); (**C**) Eye survival curves of HPG-RBG1 CDXs and the reference model Y79 (commercial cell line with *MYCN*${}_{ampl}$) for *n* = 10–11 animals per group (log-rank test, *p* < 0.05); (**D**) Tumor dissemination pattern in Y79 and HPG-RBG1 CDXs (*n* = 10-11 in each group) by RT-qPCR; (**E**) Experimental scheme design on the basis of in vitro sensitivity obtained by HTS and previous reports of pharmacologically attainable concentrations; (**F**) Eye survival curves of HPG-RBG1 after two cycles of vehicle, standard chemotherapy (carboplatin 34 mg/kg, ip), or the proposed chemotherapy treatment scheme for *n* = 5 animals per group (log-rank test, *p* < 0.05).

We developed an orthotopic cell line derived xenograft (CDX) after intravitreal injection of HPG-RBG1 cells to evaluate the efficacy of the drug combination (Figure 6A). This model resembled the histopathological features observed in Patient 2 (Figure 6B) and behaved as an aggressive tumor growth with a significant lower median eye survival of 29 days when compared to 35 days obtained in the CDXs established from Y79 (*p* < 0.05, Figure 6C). Of note, the dissemination pattern of Patient 2 was closely recapitulated by the CDXs because they showed cervical lymph node involvement without systemic (bone marrow and blood) and CNS (brain) tumor metastasis. In contrast, 73% of Y79 CDXs developed brain dissemination (Fisher’s exact test, *p* = 0.001), all optic nerves were infiltrated (Fisher’s exact test *p* = 0.0074), and all lymph nodes were free of tumor (Fisher’s exact test *p* = 0.0002) as compared to 0%, 64%, and 80% brain, optic nerve, and lymph node infiltration in HPG-RBG1 CDXs, respectively (Figure 6D).

Subsequently, the established CDXs were used to compare the in vivo efficacy of the combination of carboplatin-panobinostat-bortezomib to carboplatin as the standard of care widely used in the clinic of retinoblastoma. Doses and schedule of treatment (Figure 6E) with triple therapy were well tolerated by the animals and provided a significant eye survival advantage as compared to vehicle and carboplatin treated mice (median survival 69 days versus 41 days, log-rank test *p* < 0.01) as shown in Figure 6F. Three eyes treated with the triple scheme were free of tumor up to 100 days post-treatment. Notably, 80% of HPG-RBG1 CDXs treated with carboplatin and 100% of those that received vehicle showed tumor dissemination in the cervical lymph nodes (*p* > 0.05, Fisher’s exact test). On the contrary, all of the lymph nodes of the CDXs treated with the triple scheme of carboplatin-panobinostat-carboplatin were free of tumor at the end of treatment, as measured by RT-qPCR (*p* < 0.05, when compared to lymph node infiltration of both vehicle- and carboplatin-treated animals).

## 3. Discussion

*MYCN*${}_{ampl}$*RB1*${}^{+/+}$ patients are a rare subgroup of retinoblastomas comprising young patients with unilateral disease and aggressive histopathological features. We report for the first time a comprehensive genomic, clinical, and histopathological analysis of two highly aggressive *MYCN*${}_{ampl}$*RB1*${}^{+/+}$ metastatic retinoblastomas.

Extraocular dissemination of retinoblastoma is uncommon in high-income countries. However, our cases came from low- and middle-income countries with late diagnosis and difficulties with the family acceptance of enucleation [5,21,22,23]. In one of our cases, the local team attempted eye salvage and his outcome is unclear should he have received a primary enucleation. Our cases may indicate that these patients should not be considered for salvage treatment. However, because there are currently no specific clinical patterns aiding for the differential diagnosis of *MYCN*${}_{ampl}$*RB1*${}^{+/+}$ cases, it would be difficult to clinically identify this subtype. Access to aqueous humor sampling could provide the tool to genotype and tailor therapy in these patients [24,25]. Of note, our patients were diagnosed at 17 and 30 months, while the median age at diagnosis previously reported was 4.5 months [6]; thus, it is possible that delay in diagnosis played a role as a determinant for extraocular dissemination, potentially allowing for the acquisition of additional genomic features, as reported for *RB1*${}^{-/-}$ tumors [24].

Contrary to the common dissemination pattern of *RB1*${}^{-/-}$ cases, in which the tumor usually spreads through the optic nerve or disseminates hematogenously and progress with central nervous system invasion or bone marrow disease, our two patients showed a distinct dissemination pattern favoring massive orbital invasion, but lacked overt CNS invasion [26,27]. Pre-auricular lymph node involvement may be occasionally seen in orbital retinoblastoma with tumor infiltration to the conjunctiva, the only anatomical route to gain access from the eye to regional lymph nodes [28]. However, Patient 2 showed bulky lymph node involvement, with additional compromise of cervical and infra-diaphragmatic lymph nodes that is distinctly uncommon for retinoblastoma suggesting a particular behavior. Both patients also had subsequent systemic dissemination, including massive liver involvement, soft tissue metastasis, but no CNS invasion, even at terminal stages. Even though most current treatment protocols call for the use of neo-adjuvant chemotherapy followed by surgery in cases of orbital retinoblastoma, this regional dissemination pattern together with the lack of chemosensitivity to common chemotherapeutic agents would make it to consider initial radical surgery as a possible upfront treatment for cases with massive orbital involvement and *MYCN*${}_{ampl}$*RB1*${}^{+/+}$. In Patient 1, radical but delayed surgery was done, although it did not prevent systemic dissemination.

We established a CDX model from HPG-RBG1 cells in order to evaluate the dissemination pattern of Patient 2. This model developed an aggressive ocular tumor characterized by an engraftment efficiency of 100% and a significantly shorter eye survival as compared to Y79 CDXs. Additionally, the model recapitulated the clinical behavior of Patient 2, as almost all of HPG-RBG1 xenografts showed lymph node infiltration without brain involvement. In contrast, Y79 CDXs showed a significant higher frequency of optic nerve and brain infiltration, as expected from previous reports and based on the proximity of the anatomical structures, but the lymph nodes were free of tumor [26,29]. Lymph node dissemination was previously reported in two transgenic mice models with *RB1*${}^{-/-}$ retinoblastoma and *MYCN* amplification [30]. Thus, we provided further experimental evidence for the lymph node site-specific dissemination pattern of our case. Nonetheless, it is uncertain whether this would be a feature of other cases with *MYCN*${}_{ampl}$*RB1*${}^{+/+}$. The lack of systemic dissemination observed in the CDXs may have resulted from the lack of hematogenous dissemination and premature ending of ocular tumor growth due to ethical compliance.

Common genomic features of both cases included *MYCN* high amplification and losses in chromosome 16q and 17p (although in a subclonal state in Patient 1). For Patient 2, the loss of 17p included the selection of a mutant version of *TP53*. Mutations in *TP53* gene or in other genes implicated in this pathway are among the most frequent tumorigenic pathways in several pediatric malignancies [31,32,33,34]. In *RB1*${}^{-/-}$ retinoblastoma, amplification and high expression of *TP53* negative regulators *MDM4* and *MDM2* and loss of the MDM2 inhibitor p14ARF have been proposed as alternative mechanisms of p53 inactivation of its tumor suppresor function as the genetic inactivation of *TP53* itself were only reported anecdotally [2,5,35,36,37,38,39,40]. For *MCYN*${}_{ampl}$*RB1*${}^{+/+}$ cases, information about the status of *TP53* is unavailable [6]. Nonetheless, our second case showed a somatic mutation in LOH in *TP53* gene in the treated primary tumor and the metastatic sites, without accompanying amplifications in upstream regulators. The relation between *TP53* oncogenic function and tumor aggressiveness in the context of *MCYN*${}_{ampl}$*RB1*${}^{+/+}$ should be further explored.

The presence of the *TP53* mutation was coincident with a high chromosomal instability in the specimens of Patient 2. The highly heterogeneous and aneuploid metastatic samples of Patient 2 are remarkable, as usually retinoblastomas characteristically present diploid tumors [2]. In addition, *MCYN*${}_{ampl}$*RB1*${}^{+/+}$ have been described as having fewer copy-number alterations compared to *RB1*${}^{-/-}$ tumors [6]. It is well known that the loss of RB1 results in mitotic defects that can lead to aneuploidy [41]; this case, however, has an intact pRb function. The presence of a potentially damaging *TP53* mutation in homozygosis due to the 17p in LOH may be responsible for the chromosomal instability phenotype, as there is ample evidence that the *TP53* pathway is a major contributor to genome instability in adult and pediatric cancer [42,43,44]. Interestingly, the manual review of the bioinformatic analysis along with the karyotype analysis of HPG-RBG1 cells concluded that the metastatic samples were near-triploid for the lymph node and near-tetraploid for the orbital tumor. Whole genome doubling (WGD), present in almost one-third of human cancers, is associated with aneuploidy and a subsequently increased loss of chromosomes [45]. This is the most likely event that, followed by consecutive chromosomal losses, led to a near-tri or tetraploid state in metastatic samples of Patient 2. Additionally, as the *TP53* mutation and 16q and 17p loss are in LOH in those samples, these genomic events might have occurred previous to WGD.

*MYCN* amplification along with *TP53* inactivation results in an aggressive phenotype seen in other pediatric embryonal tumors such as relapsed medulloblastoma [46]. The emergence of a combined *TP53* mutation and *MYCN* amplification at relapse proved to be a feature that occurs irrespective of the medulloblastoma subgroup, involves specific combinations of events not observed at diagnosis, and it is associated with rapid progression to death [46]. In Patient 2 samples, the evidence of a transition from an unstable CNA profile in the treated, primary tumor to a highly aneuploid genome in the metastatic sites suggest a similar behavior for this case of retinoblastoma. Our data supports a model of evolution in which an heterogeneous *MYCN*${}_{ampl}$*RB1*${}^{+/+}$ primary tumor presented a subclonal oncogenic *TP53* mutation that was probably selected and amplified by chemotherapy, further stabilized by LOH [47], which generated chromosomal instability and WGD, with later losses of genetic material and/or chromothrypsis. In the metastasis, as suggested by the fewer CNAs observed in the orbit with respect to the lymph node samples and the divergence of somatic mutations, the dissemination niche also contributed to further evolution. This interpretation is consistent with the recently published evidence that different pediatric solid cancers relapses share a common generic pattern of clonal expansion and evolution of somatic genomic changes, in which high risk aberrations, such as *MYCN* amplification in neuroblastoma and *TP53* in Wilms tumor lead to complex chromosome changes and anaplastic histological features [48]. Other aberrations, such as the *OTX2* amplification in the lymph node sample [11,49] and the 11p deletion in the orbital metastasis (aberration mainly described in Wilms tumors in children [50]), may be contributing to the phenotypes, although no clear association could be established. We can also speculate that a similar phenomenon may have happened in Patient 1 given the coincidences in histopathological features and the lack of response to standard therapy. The late diagnosis, the lack of specimens for genomic analysis and the short clinical evolution prevented a precise evaluation of this case.

On the contrary, neuroblastoma, a tumor with neural origin like retinoblastoma seems to behave differently as in *MYCN*-amplified neuroblastomas diploidy is associated with a poorer outcome than hyperdiploidy [51]. Moreover, although neuroblastoma can evolve to a more aggressive phenotype with mixed numerical (i.e. chromosomal) and segmental (i.e. arm or shorter segments) profile, as seen in Patient 2, this has not been shown to occur in *MYCN*-amplified neuroblastoma (20–25% of all neuroblastomas) [52].

Both of our patients had tumors that progressed under standard therapy. In line with this observation, cells derived from the lymph node metastasis were between 250-fold and 3.6-fold less sensitive (higher IC50) to standard chemotherapy used for treatment of this patient as compared to the cells derived from the intraocular tumor after upfront enucleation (Table S4). These cells as well as the matched tumor also harbor a *TP53* mutation and, thus, the low sensitivity to chemotherapy may be the result of MYCN amplification, aneuploidy, *TP53* somatic mutation, or due to combinations of aberrations. Nonetheless, they may not represent the sensitivity pattern of other *MYCN*${}_{ampl}$*RB1*${}^{+/+}$ cells without additional *TP53* mutation. Only 17 compounds fulfilled the selection criteria for potential translation into the clinics. Most of the active agents were HDAC inhibitors. Due to the epigenetic modulation, these agents offer an effective means to therapeutically alter the regulation of proto-oncogenes and tumor suppressor genes in malignant cells and induce pro-apoptotic transcriptional changes in *MYCN* amplified neuroblastoma cell lines [53,54]. Also, bortezomib was retained in the selection process as active against HPG-RBG1 cells. The proteasome inhibitor alters cellular protein homeostasis and has shown to prevent *MYCN* degradation resulting in its accumulation with a deleterious effect in *MYCN*-driven neuroblastomas [55,56]. Bortezomib was also selected based on single-agent activity and promising features in combination studies in pediatric preclinical models [20,57,58].

Several compounds active against HPG-RBG1 were excluded for further analysis due to unavailability of clinical evidence of pediatric use or testing (e.g., STAT3 and survivin inhibitors), the lack of widely available drugs (e.g., ceritinib, fimepinostat), or because active concentrations would not be clinically achievable based on the current clinical doses and data reported from previous pharmacokinetic studies for a rapid clinical translation [59]. Of note, bromodomain and extraterminal (BET) inhibitors exerted much less than 80% inhibition of HPG-RBG1 cell growth at the maximal evaluated concentration. The inhibition of BET proteins affects transcriptional regulation of several oncogenes, including *MYCN* inducing cell cycle arrest and apoptosis in preclinical models of neuroblastoma with *MYCN*${}_{ampl}$. However, the lack of cytotoxicity being exerted in our cell model may be explained by the fact that BET inhibitor activity depends on wild-type *TP53* and, thus, may not exert its full activity in our cell model [60]. Lastly, another approach could be to target aneuploidy by means of proteotoxicity due to cellular stress [45]. The protein quality control machinery is restricted in aneuploid cells limiting their degradation capacity and, thus, explaining the activity of several compounds belonging to the family of heat shock protein 90 (HSP90) against HPG-RBG1 cells (Table S3). Unfortunately, reports on the retinal toxicity of HSP90 inhibitors as well as systemic toxicities may hinder the use in retinoblastoma [61,62].

Thereafter, drugs were evaluated in combinations in order to identify those with synergistic cytotoxic effects. Despite the low in vitro cytotoxic activity that was predicted by the established association between platinum resistance and *TP53* mutations in other tumors [63,64], carboplatin was included due to the long experience in the use of these agents in metastatic retinoblastoma treatment [20]. The most promising in vitro cytotoxic combinations were carboplatin with panobinostat and panobinostat with bortezomib and, likewise, the triple-drug combination significantly prolonged eye survival in HPG-RBG1 xenograft relative to standard of care carboplatin supporting the potential translation to the clinics of the pharmacological combination. Of note, lymph node dissemination was prevented in all animals treated with the triple-drug combination in contrast to those that received only carboplatin as the standard-of-care supporting the use of HDACi and proteasome inhibitors as part of the adjuvant chemotherapy in these cases. Lastly, in one of our cases studied, membrane expression of the ganglioside GD2 was evident, so the use of anti-GD2 immunotherapy as in neuroblastoma may be considered given the limited therapeutic options for these cases [65].

## 4. Materials and Methods

### 4.1. Ethics Statement

Informed consent has been properly obtained from parents or guardians.

### 4.2. Immunohistochemistry, Immunofluorescence, And Fluorescent In Situ Hybridization

Immunohistochemistry was performed in FFPE tissues and in fixed primary cell cultures. The following antibodies and manufacturers were used Arrestin 3 (11100-2-AP, Proteintech group, Rosemont, IL, USA), synaptophysin (NCL-L-SYNAP-299, Leica, Buffalo Grove, IL, USA), pRb (NCL-L.RB358, Leica, Buffalo Grove, IL, USA), CRX (ab14603, Abcam, Cambridge, MA, USA), p53 (DO-7, Leica, Buffalo Grove, IL, USA), and Ki67 (M7240, Agilent, Santa Clara, CA, USA) under the conditions that were recommended by the manufacturer. GD2 detection was performed by immunofluorescence using the 3F8 antibody [8]. *MYCN* amplification was assessed by FISH, as described in Supplementary Materials.

### 4.3. Rb1 Alterations, Whole-Exome Sequencing and Oncoscan Copy Number Variation Analysis

RB1 mutation analysis was performed, as previously reported [66]. For Patient 2, we also analyzed RB1 promoter methylation status using SALSA MLPA probemix ME002-C1 Tumour suppressor mix 2 (MRC-Holland). For FFPE samples of the primary tumor of patients, genome-wide DNA copy number alterations and allelic imbalances were analyzed using the Affymetrix OncoScan platform after DNA extraction using PureLink Genomic DNA mini kit (Invitrogen, Carlsbad, CA, USA). Array CEL files were processed using the OncoScan Console Software (Thermo Fisher Scientific, Waltham, MA, USA) and they were visualized with Nexus Expression OncoScan software (BioDiscovery, El Secondo, CA, USA), Chromosome Analysis Suite (Thermo Fisher Scientific, Waltham, MA, USA), and a custom script. Based on the comparison with calls from a normal cohort and the database of benign copy number variants, copy number aberrations greater than 500 kb in size, and copy-neutral LOH greater than 10 Mb are considered to be abnormal based on the established performance characteristics of the assay. For the orbital and lymph node metastasis biopsies of Patient 2 fresh tumors were available for whole exome sequencing (WES). Exome capture was performed by SureSelect Clinical Research Exome v2 (Agilent Technologies, Santa Clara, CA, USA) followed by Next Generation Sequencing on an Illumina HiSeq 4000 instrument (Illumina, San Diesgo, CA, USA). The bioinformatic analysis was performed, as described in Supplementary Materials.

Somatic CNA analysis from aligned BAM files was performed using Control-FREEC [67] for WES with default parameters, except for ploidy which was set to 3. The DbSNP database was used for BAF calculations [68]. Manual review of the results, using the CNA table, LRR, and BAF profiles, was done for all of the samples. In the orbit sample, manual reformulation of CN status was done, as ploidy was near-tetraploid in this case.

### 4.4. Establishment and Characterization of Tumor Cell Line

Patient-derived primary cell culture was established after fine needle aspiration of the cervical lymph node of Patient 2. The fresh tumor sample was mechanically disaggregated and cultured in neural stem-cell medium, as previously described [29]. After three passages, the cell culture was considered to be established and named after the code RBG-HPG1. For comparison, we also included the commercial cell line Y79 (ATCC, HTB-18) and a primary cell culture derived from an intraocular tumor after upfront enucleation (HPG-RBT-12) previously reported [16,69]. All cultures were maintained at 37 °C in 5% CO2 atmosphere.

For HPG-RBG1, cell line authentication was performed by short tandem repeat (STR) profiling. Subsequently, WES was performed on an Illumina HiSeq 4000 and chromosomal aberrations (CNAs and mutations) were compared to the genomic profile of the matched-tumor.

CRX expression was analyzed in ordr to confirm the retinal tumor origin after RNA isolation using PureLink RNA Mini Kit (Invitrogen, Carlsbad, CA, USA) by means of real-time quantitative PCR (RT-qPCR), as described [7,29]. Immunohistochemistry of FFPE cells was performed for morphological assessment (hematoxylin-eosin), retinal (arrestin3, CRX), and neuroectodermic (synaptophysin) tumor markers, markers of cell proliferation (Ki67), and to assess for the expression pRb (Supplementary Materials).

### 4.5. Drug Screening, In Vitro and In Vivo Pharmacological Evaluations

After establishing the optimal high-throughput drug screening (HTS) assay conditions were as described elsewhere, we assessed large-scale drug sensitivity against the tumor cell lines, as described in Supplementary Materials.

We then performed a primary compound selection and thereafter a full concentration-response curves (full description in Supplementary Materials) for those compounds that achieved the following criteria: >80% inhibition of cell growth in the primary screen; FDA-approved drugs; available reports of Phase I/II trials in pediatrics or drugs currently used in pediatric oncology (Figure S7). Thereafter, drug combination analysis was performed in order to evaluate potentially synergistic effects among standard of care and the selected repurposing drugs for ease in the clinical translation of the findings (Supplementary Materials).

### 4.6. Establishment, Characterization and Pharmacological Evaluation in a Patient-Derived Xenograft Model of *MYCN*${}_{ampl}$ *RB1*${}^{+/+}$

Animal studies were performed in accordance to the tenets of Association for Research in Vision and Ophthalmology for the use of Animals in Ophthalmic and Vision Research and were approved by the Institutional Animal Care and Use Committee (IACUC) of Fundación Instituto Leloir (protocol # 2019-069).

Full description of the procedures for CDX establishment, evaluation of tumor dissemination, and ocular survival is provided in Supplementary Materials as well as in previous reports [29].

Subsequently, drug combinations consisting of carboplatin, panobinostat, and bortezomib were evaluated for antitumor response as compared to carboplatin as the standard of care (Supplementary Materials).

## 5. Conclusions

Altogether, the present cases confirm the aggressive biology of of *MYCN*${}_{ampl}$*RB1*${}^{+/+}$ with one case showing additional *TP53* somatic mutation in a context of high aneuploidy. These cases showed a common dissemination pattern, including loco-regional, lymph node, and systemic metastasis with dismal outcome and poor sensitivity to standard chemotherapy drugs. Carboplatin, panobinostat, and bortezomib combination were identified as possible candidate treatments that are based on in vitro and in vivo preclinical assessments.

## Supplementary Materials

The following are available online at https://www.mdpi.com/2072-6694/12/9/2714/s1. Supplementary Materials, Figure S1: P53 staining of tumor samples from patients, Figure S2: Log-ratio (LRR) and B-Allele Frequency (BAF) profile of Patient 1 ocular tumor, Figure S3: Log-ratio (LRR) and B-Allele Frequency (BAF) profile of Patient 2 primary ocular tumor, Figure S4: Characterization of HPG-RBG1 cells, Figure S5: Log-ratio (LRR) and B-Allele Frequency (BAF) profiles of patient 2 orbital and lymph-node metastasis, and its derived cell line (HPG-RBG1), Figure S6: Flowchart depicting the decision selection criteria for drug screening, Table S1: Short tandem repeat analysis of HPG-RBG1 cells and paired tumor, Table S2: Somatic mutation analysis for Patient 1 and 2 samples, Table S3: List of active compounds with cytotoxic activity of at least 80% in HPG-RBG1 cells, Table S4: Drug sensitivity for active compounds that passed to secondary screen and comparison of the sensitivity to selected active compounds among retinoblastoma cell lines.

## Abbreviations

The following abbreviations are used in this manuscript:

BAF	B-Allele frequency
CDX	Cell line derived xenograft
CNA	Copy number alteration
CNS	Central nervous system
COG-ARET0321	Children’s Oncology Group trial (ARET0321)
CSF	Cerebrospinal fluid
CT	Computerized tomography
FISH	Fluorescence in situ hybridization
HPG-RBG1	Lymph node metastasis-derived cell line
HTS	High throughput screening
LOH	Loss of heterozygosity
LRR	Log-ratio (Base-2 logarithmic ratio)
MLPA	Multiplex ligation-dependent probe amplification
MRI	Magnetic resonance imaging
*MYCN*${}_{ampl}$	*MYCN* amplified
*RB1*${}^{+/+}$	wild-type *RB1*
*RB1*${}^{-/-}$	aberrant *RB1*
SEM	Standard error of the mean
STR	Short tandem repeat
WES	Whole exome sequencing
WGD	Whole genome doubling

## References

[1] Knudson A.G. (1971). Mutation and cancer: Statistical study of retinoblastoma. Proc. Natl. Acad. Sci. USA.

[2] Corson T.W., Gallie B.L. (2007). One hit, two hits, three hits, more? Genomic changes in the development of retinoblastoma Genes Chromosom. Cancer.

[3] Dimaras H., Khetan V., Halliday W., Orlic M., Prigoda N.L., Piovesan B., Marrano P., Corson T.W., Eagle R.C., Squire J.A. (2008). Loss of RB1 induces non-proliferative retinoma: Increasing genomic instability correlates with progression to retinoblastoma. Hum. Mol. Genet..

[4] Singh H.P., Wang S., Stachelek K., Lee S., Reid M.W., Thornton M.E., Craft C.M., Grubbs B.H., Cobrinik D. (2018). Developmental stage-specific proliferation and retinoblastoma genesis in RB-deficient human but not mouse cone precursors. Proc. Natl. Acad. Sci. USA.

[5] Munier F.L., Beck-Popovic M., Chantada G.L., Cobrinik D., Kivelä T.T., Lohmann D., Maeder P., Moll A.C., Carcaboso A.M., Moulin A. (2019). Conservative management of retinoblastoma: Challenging orthodoxy without compromising the state of metastatic grace. “Alive, with good vision and no comorbidity”. Prog. Retin. Eye Res..

[6] Rushlow D.E., Mol B.M., Kennett J.Y., Yee S., Pajovic S., Thériault B.L., Prigoda-Lee N.L., Spencer C., Dimaras H., Corson T.W. (2013). Characterisation of retinoblastomas without RB1 mutations: Genomic, gene expression, and clinical studies. Lancet Oncol..

[7] Torbidoni A.V., Laurent V.E., Sampor C., Ottaviani D., Vazquez V., Gabri M.R., Rossi J., de Dávila M.T., Alonso C., Alonso D.F. (2015). Association of Cone-Rod Homeobox Transcription Factor Messenger RNA With Pediatric Metastatic Retinoblastoma. JAMA Ophthalmol..

[8] Laurent V.E., Torbidoni A.V., Sampor C., Ottaviani D., Vazquez V., Gabri M.R., Garcia De Davila M.T., Ramirez-Ortiz M.A., Alonso C.N., Rossi J. (2016). Minimal disseminated disease in no nMetastatic retinoblastoma with high-risk pathologic features and association with disease-free survival. JAMA Ophthalmol..

[9] Laurent V.E., Sampor C., Solernou V., Rossi J., Gabri M., Eandi-Eberle S., De Davila M.T., Alonso D.F., Chantada G.L. (2013). Detection of minimally disseminated disease in the cerebrospinal fluid of children with high-risk retinoblastoma by reverse transcriptase-polymerase chain reaction for GD2 synthase mRNA. Eur. J. Cancer.

[10] Chantada G.L., Rossi J., Casco F., Fandiño A., Scopinaro M., De Dávila M.T.G., Abramson D.H. (2006). An aggressive bone marrow evaluation including immunocytology with GD2 for advanced retinoblastoma. J. Pediatr. Hematol..

[11] McEvoy J., Nagahawatte P., Finkelstein D., Richards-Yutz J., Valentine M., Ma J., Mullighan C., Song G., Chen X., Wilson M. (2014). RB1 gene inactivation by chromothripsis in human retinoblastoma. Oncotarget.

[12] Landrum M.J., Lee J.M., Benson M., Brown G.R., Chao C., Chitipiralla S., Gu B., Hart J., Hoffman D., Jang W. (2018). ClinVar: Improving access to variant interpretations and supporting evidence. Nucleic Acids Res..

[13] Kopanos C., Tsiolkas V., Kouris A., Chapple C.E., Albarca Aguilera M., Meyer R., Massouras A. (2019). VarSome: The human genomic variant search engine. Bioinformatics.

[14] Dimaras H., Corson T.W., Cobrinik D., White A., Zhao J., Munier F.L., Abramson D.H., Shields C.L., Chantada G.L., Njuguna F. (2015). Retinoblastoma. Nat. Rev. Dis. Prim..

[15] Zhang J., Benavente C.A., McEvoy J., Flores-Otero J., Ding L., Chen X., Ulyanov A., Wu G., Wilson M., Wang J. (2012). A novel retinoblastoma therapy from genomic and epigenetic analyses. Nature.

[16] Winter U., Ganiewich D., Ottaviani D., Zugbi S., Aschero R., Sendoya J.M., Cafferata E.G., Mena M., Sgroi M., Sampor C. (2020). Genomic and transcriptomic tumor heterogeneity in bilateral retinoblastoma. JAMA Ophthalmol..

[17] Van Veggel M., Westerman E., Hamberg P. (2018). Clinical Pharmacokinetics and Pharmacodynamics of Panobinostat. Clin. Pharmacokinet..

[18] Wood P.J., Strong R., McArthur G.A., Michael M., Algar E., Muscat A., Rigby L., Ferguson M., Ashley D.M. (2018). A phase I study of panobinostat in pediatric patients with refractory solid tumors, including CNS tumors. Cancer Chemother. Pharmacol..

[19] Hennika T., Hu G., Olaciregui N.G., Barton K.L., Ehteda A., Chitranjan A., Chang C., Gifford A.J., Tsoli M., Ziegler D.S. (2017). Pre-clinical study of panobinostat in xenograft and genetically engineered murine diffuse intrinsic pontine glioma models. PLoS ONE.

[20] Stewart E., Federico S.M., Chen X., Shelat A.A., Bradley C., Gordon B., Karlstrom A., Twarog N.R., Clay M.R., Bahrami A. (2017). Orthotopic patient-derived xenografts of paediatric solid tumours. Nature.

[21] Chawla B., Hasan F., Seth R., Pathy S., Pattebahadur R., Sharma S., Upadhyaya A., Azad R. (2016). Multimodal Therapy for Stage III Retinoblastoma (International Retinoblastoma Staging System): A Prospective Comparative Study. Ophthalmology.

[22] Leal-Leal C.A., Rivera-Luna R., Flores-Rojo M., Juárez-Echenique J.C., Ordaz J.C., Amador-Zarco J. (2006). Survival in extra-orbital metastatic retinoblastoma: Treatment results. Clin. Transl. Oncol..

[23] Canturk S., Qaddoumi I., Khetan V., Ma Z., Furmanchuk A., Antoneli C.B., Sultan I., Kebudi R., Sharma T., Rodriguez-Galindo C. (2010). Survival of retinoblastoma in less-developed countries impact of socioeconomic and health-related indicators. Br. J. Ophthalmol..

[24] Polski A., Xu L., Prabakar R.K., Gai X., Kim J.W., Shah R., Jubran R., Kuhn P., Cobrinik D., Hicks J. (2020). Variability in retinoblastoma genome stability is driven by age and not heritability. Genes Chromosom. Cancer.

[25] Berry J.L., Xu L., Murphree A.L., Krishnan S., Stachelek K., Zolfaghari E., McGovern K., Lee T.C., Carlsson A., Kuhn P. (2017). Potential of aqueous humor as a surrogate tumor biopsy for retinoblastoma. JAMA Ophthalmol..

[26] Chevez-Barrios P., Hurwitz M.Y., Louie K., Marcus K.T., Holcombe V.N., Schafer P., Aguilar-Cordova C.E., Hurwitz R.L. (2000). Metastatic and no nMetastatic models of retinoblastoma. Am. J. Pathol..

[27] McClean I., Burnier M., Zimmerman L.J.F. (1994). Tumors of the retina. Tumors of the eye and adnexa. Atlas of Tumor Pathology. Am. J. Surg. Pathol. Febr..

[28] Honavar S.G., Manjandavida F.P., Reddy V.A.P. (2017). Orbital retinoblastoma: An update. Rev. Artic..

[29] Pascual-Pasto G., Olaciregui N.G., Vila-Ubach M., Paco S., Monterrubio C., Rodriguez E., Winter U., Batalla-Vilacis M., Catala J., Salvador H. (2016). Preclinical platform of retinoblastoma xenografts recapitulating human disease and molecular markers of dissemination. Cancer Lett..

[30] MacPherson D., Conkrite K., Tam M., Mukai S., Mu D., Jacks T. (2007). Murine bilateral retinoblastoma exhibiting rapid-onset, metastatic progression and N-myc gene amplification. EMBO J..

[31] Donehower L.A., Soussi T., Korkut A., Liu Y., Schultz A., Cardenas M., Li X., Babur O., Hsu T.K., Lichtarge O. (2019). Integrated Analysis of TP53 Gene and Pathway Alterations in The Cancer Genome Atlas. Cell Rep..

[32] Alejandro Sweet-Cordero E., Biegel J.A. (2019). The genomic landscape of pediatric cancers: Implications for diagnosis and treatment. Science.

[33] Oh L., Hafsi H., Hainaut P., Ariffin H. (2019). P53, stem cell biology and childhood blastomas. Curr. Opin. Oncol..

[34] Patel R.R., Ramkissoon S.H., Ross J., Weintraub L. (2020). Tumor mutational burden and driver mutations: Characterizing the genomic landscape of pediatric brain tumors. Pediatr. Blood Cancer.

[35] Kato M.V., Shimizu T., Ishizaki K., Kaneko A., Yandell D.W., Toguchida J., Sasaki M.S. (1996). Loss of heterozygosity on chromosome 17 and mutation of the p53 gene in retinoblastoma. Cancer Lett..

[36] Livide G., Epistolato M.C., Amenduni M., Disciglio V., Marozza A., Mencarelli M.A., Toti P., Lazzi S., Hadjistilianou T., De Francesco S. (2012). Epigenetic and copy number variation analysis in retinoblastoma by MS-MLPA. Pathol. Oncol. Res..

[37] Guo Y., Pajovic S., Gallie B.L. (2008). Expression of p14ARF, MDM2, and MDM4 in human retinoblastoma. Biochem. Biophys. Res. Commun..

[38] Laurie N.A., Donovan S.L., Shih C.S., Zhang J., Mills N., Fuller C., Teunisse A., Lam S., Ramos Y., Mohan A. (2006). Inactivation of the p53 pathway in retinoblastoma. Nature.

[39] Castéra L., Sabbagh A., Dehainault C., Michaux D., Mansuet-Lupo A., Patillon B., Lamar E., Aerts I., Lumbroso-Le Rouic L., Couturier J. (2010). MDM2 as a modifier gene in retinoblastoma. J. Natl. Cancer Inst..

[40] Conkrite K., Sundby M., Mu D., Mukai S., MacPherson D. (2012). Cooperation between Rb and Arf in suppressing mouse retinoblastoma. J. Clin. Investig..

[41] Benavente C.A., Dyer M.A. (2015). Genetics and Epigenetics of Human Retinoblastoma. Annu. Rev. Pathol. Mech. Dis..

[42] Taylor A.M., Shih J., Ha G., Gao G.F., Zhang X., Berger A.C., Schumacher S.E., Wang C., Hu H., Liu J. (2018). Genomic and Functional Approaches to Understanding Cancer Aneuploidy. Cancer Cell.

[43] Burrell R.A., McGranahan N., Bartek J., Swanton C. (2013). The causes and consequences of genetic heterogeneity in cancer evolution. Nature.

[44] Karlsson J., Valind A., Holmquist Mengelbier L., Bredin S., Cor nMark L., Jansson C., Wali A., Staaf J., Viklund B., Øra I. (2018). Four evolutionary trajectories underlie genetic intratumoral variation in childhood cancer. Nat. Genet..

[45] Ben-David U., Amon A. (2020). Context is everything: Aneuploidy in cancer. Nat. Rev. Genet..

[46] Hill R.M., Kuijper S., Lindsey J.C., Petrie K., Schwalbe E.C., Barker K., Boult J.K., Williamson D., Ahmad Z., Hallsworth A. (2015). Combined MYC and P53 defects emerge at medulloblastoma relapse and define rapidly progressive, therapeutically targetable disease. Cancer Cell.

[47] Alexandrova E.M., Mirza S.A., Xu S., Schulz-Heddergott R., Marchenko N.D., Moll U.M. (2017). P53 loss-of-heterozygosity is a necessary prerequisite for mutant p53 stabilization and gain-of-function in vivo. Cell Death Dis..

[48] Andersson N., Bakker B., Karlsson J., Valind A., Mengelbier L.H., Spierings D.C., Foijer F., Gisselsson D. (2020). Extensive clonal branching shapes the evolutionary history of high-risk pediatric cancers. Cancer Res..

[49] Glubrecht D.D., Kim J.H., Russell L., Bamforth J.S., Godbout R. (2009). Differential CRX and OTX2 expression in human retina and retinoblastoma. J. Neurochem..

[50] Deng C., Dai R., Li X., Liu F. (2016). Genetic variation frequencies in Wilms’ tumor: A meta-analysis and systematic review. Cancer Sci..

[51] Schneiderman J., London W.B., Brodeur G.M., Castleberry R.P., Look A.T., Cohn S.L. (2008). Clinical significance of MYCN amplification and ploidy in favorable-stage neuroblastoma: A report from the Children’s Oncology Group. J. Clin. Oncol..

[52] Schleiermacher G., Janoueix-Lerosey I., Ribeiro A., Klijanienko J., Couturier J., Pierron G., Mosseri V., Valent A., Auger N., Plantaz D. (2010). Accumulation of segmental alterations determines progression in neuroblastoma. J. Clin. Oncol..

[53] Hegarty S.V., Togher K.L., O’Leary E., Solger F., Sullivan A.M., O’Keeffe G.W. (2017). Romidepsin induces caspase-dependent cell death in human neuroblastoma cells. Neurosci. Lett..

[54] Huang M., Weiss W.A. (2013). Neuroblastoma and MYCN. Cold Spring Harb. Perspect. Med..

[55] Shahbazi J., Liu P.Y., Atmadibrata B., Bradner J.E., Marshall G.M., Lock R.B., Liu T. (2016). The bromodomain inhibitor jq1 and the histone deacetylase inhibitor panobinostat synergistically reduce n-myc expression and induce anticancer effects. Clin. Cancer Res..

[56] Wang J., Jiang J., Chen H., Wang L., Guo H., Yang L., Xiao D., Qing G., Liu H. (2019). FDA-approved drug screen identifies proteasome as a synthetic lethal target in MYC-driven neuroblastoma. Oncogene.

[57] Boccadoro M., Morgan G., Cavenagh J. (2005). Preclinical evaluation of the proteasome inhbitor bortezomib in cancer therapy. Cancer Cell Int..

[58] Torres J., Regan P.L., Edo R., Leonhardt P., Jeng E.I., Rappaport E.F., Ikegaki N., Tang X.X. (2010). Biological effects of induced MYCN hyper-expression in MYCN-amplified neuroblastomas. Int. J. Oncol..

[59] Ferrario A., Luna M., Rucker N., Wong S., Lederman A., Kim J., Gomer C. (2016). Targeting survivin enhances chemosensitivity in retinoblastoma cells and orthotopic tumors. PLoS ONE.

[60] Henssen A., Thor T., Odersky A., Heukamp L., El-Hindy N., Beckers A., Speleman F., Althoff K., Schäfers S., Schramm A. (2013). BET bromodomain protein inhibition is a therapeutic option for medulloblastoma. Oncotarget.

[61] Garcia-Carbonero R., Carnero A., Paz-Ares L. (2013). Inhibition of HSP90 molecular chaperones: Moving into the clinic. Lancet Oncol..

[62] Wu W.C., Wu M.H., Chang Y.C., Hsieh M.C., Wu H.J., Cheng K.C., Lai Y.H., Kao Y.H. (2010). Geldanamycin and its analog induce cytotoxicity in cultured human retinal pigment epithelial cells. Exp. Eye Res..

[63] Lorenzon I., Pellarin I., Pellizzari I., D’Andrea S., Belletti B., Sonego M., Baldassarre G., Schiappacassi M. (2019). Identification and Characterization of a New Platinum-Induced TP53 Mutation in MDAH Ovarian Cancer Cells. Cells.

[64] Boyault S., Drouet Y., Navarro C., Bachelot T., Lasset C., Treilleux I., Tabone E., Puisieux A., Wang Q. (2012). Mutational characterization of individual breast tumors: TP53 and PI3K pathway genes are frequently and distinctively mutated in different subtypes. Breast Cancer Res. Treat..

[65] Wang H., Yang J., Pan H., Tai M.C., Maher M.H., Jia R., Ge S., Lu L. (2020). Dinutuximab synergistically enhances the cytotoxicity of natural killer cells to retinoblastoma through the perforin-granzyme B pathway. Oncotargets Ther..

[66] Parma D., Ferrer M., Luce L., Giliberto F., Szijan I. (2017). RB1 gene mutations in Argentine retinoblastoma patients. Implications for genetic counseling. PLoS ONE.

[67] Boeva V., Popova T., Bleakley K., Chiche P., Cappo J., Schleiermacher G., Janoueix-Lerosey I., Delattre O., Barillot E. (2012). Control-FREEC: A tool for assessing copy number and allelic content using next-generation sequencing data. Bioinformatics.

[68] Sherry S.T., Ward M., Sirotkin K. (1999). dbSNP - database for single nucleotide polymorphisms and other classes of minor genetic variation. Genome Res..

[69] Winter U., Aschero R., Fuentes F., Buontempo F., Zugbi S., Sgroi M., Sampor C., Abramson D.H., Carcaboso A.M., Schaiquevich P. (2019). Tridimensional Retinoblastoma Cultures as Vitreous Seeds Models for Live-Cell Imaging of Chemotherapy Penetration. Int. J. Mol. Sci..

