# Supplementary material for: Clinical, Genomic, and Pharmacological Study of MYCN-Amplified RB1 Wild-Type Metastatic Retinoblastoma
Source: Cancers (Basel). 2020 Sep 22;12(9):2714. doi: 10.3390/cancers12092714 (PMC7565107; doi:10.3390/cancers12092714)
Supplement: Supplementary file 1 [file cancers-12-02714-s001.zip › Zugbi2020-supplementary-material/Zugbi2020-supplementary.zip!/SuppFigure6.pdf]

Supplementary Figure S6

A)

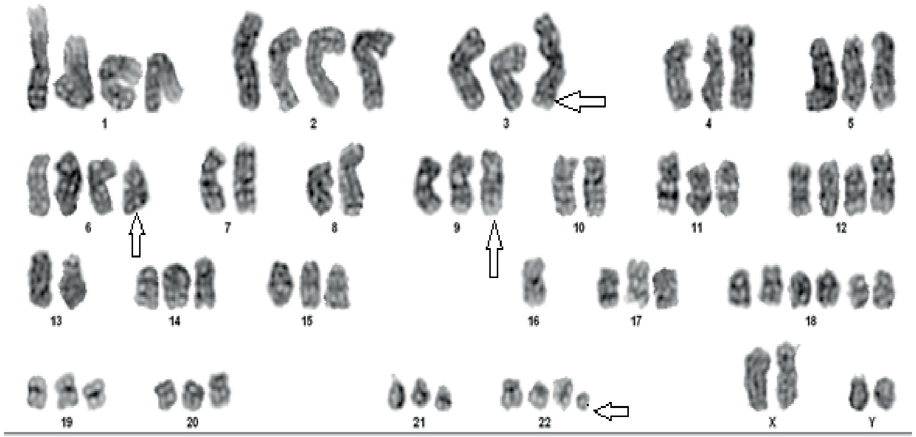

72<2n>, XY, +X, +Y, +1, +1, +2, +2, +add(3)(q27), +4, +5, +6, +?i(6)(p10), +9, ?t(9;22)(q34;q11), +11, +12, +12, +14, +15 ,-16, +17, +18, +18, +18, +18, +19, +20, +21, +22, +22

B)

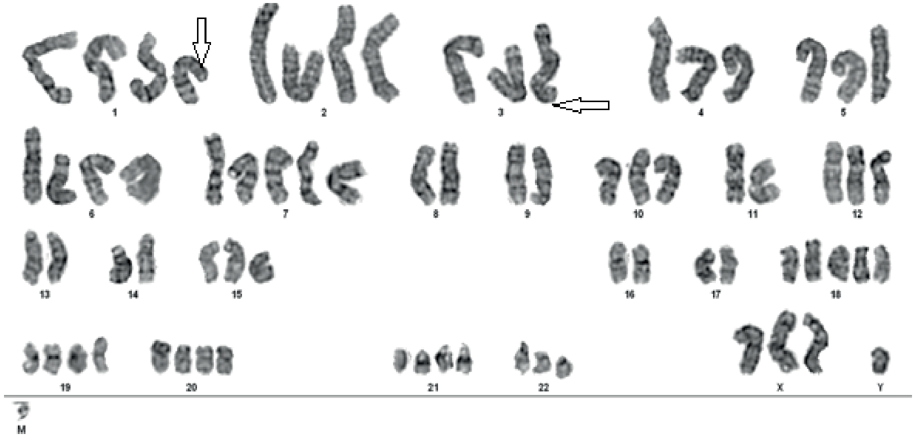

74<2n>, XY, +X, +X, +1, +add(1)(p34), +2, +2, +add(3)(q27), +4, +5, +6, +6, +7, +7, +7, +10, +12, +15, +18, +18, +18, +19, +19, +20, +20, +21, +21, +22, +dmin

C)

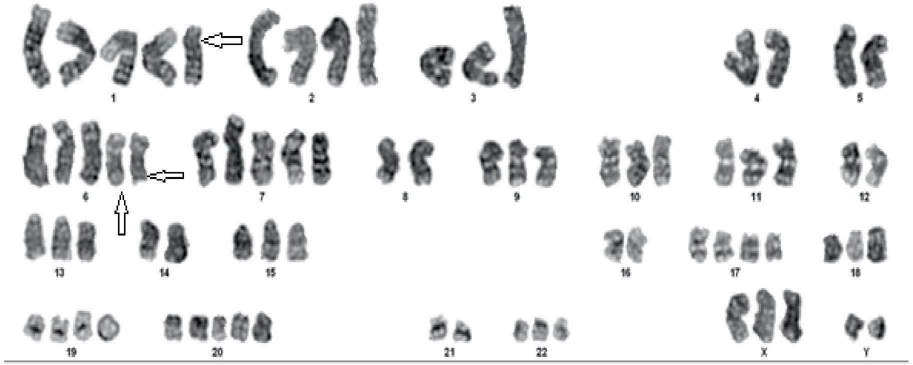

75<2n>, XY, +X, +X, +Y, +1, +1, +del(1)(p34), +2, +2, +3, +6, +del(6)(q?), +del(6)(q?), +7, +7, +7, +9, +10, +11, +13, +15, +17, +17, +18, +19, +der(19), +20, +20, +20, +22

D)

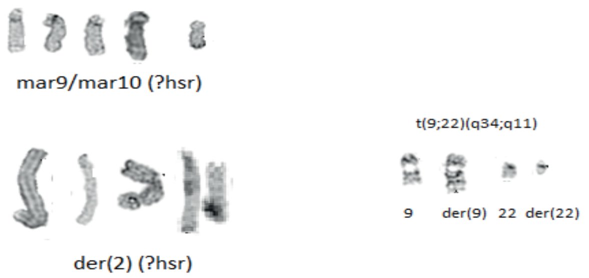

Composite kayrotype

61-76<2n>, XY, +X[7], +X, +X[9], +Y[2], +1[5], +1, +1[9], +1, +1, +1, +1[2], der(1)[3], del(1)(p34)[3], +2[1], +2, +2[6], der(2)?(hsr)[6], +3[6], +3, +3[2], add(3)(q27)[4], +4[9], +5[8], +5, +5[2]. +6[7], +6, +6[3], +del(6)(q?)[2], +del(6)(q?)x2[2], +i(6)(p10)[2], +7[2], +7, +7[7], +7, +7, +7[5], +8[8], +9[7], +9, +9[2], ?t(9;22)(q34;q11)[5], +10[6], +10, +10[2], +11[8], +11, +11[3], +12[6], +12, +12[4], +13[6], +14[5], +15[10], +16[4], +17[9], +17, +17[4], +18[9], +18, +18[2], 19[6], +19, +19[2], +20[3], +20, +20[7], +21[8], +21, +21[5], +22[11], +22, +22[4], +mar1-8, 11-23, +mar9(?hsr)[3], +mar10(?hsr)[2]+dmin[3]{cp18}
